# Recommendations for the management of tyrosinaemia type 1

**DOI:** 10.1186/1750-1172-8-8

**Published:** 2013-01-11

**Authors:** Corinne de Laet, Carlo Dionisi-Vici, James V Leonard, Patrick McKiernan, Grant Mitchell, Lidia Monti, Hélène Ogier de Baulny, Guillem Pintos-Morell, Ute Spiekerkötter

**Affiliations:** 1 Nutrition and Metabolism Unit, Department of Pediatrics, University Children’s Hospital Queen Fabiola, Brussels, Belgium; 2 Division of Metabolism, Department of Pediatric Medicine, Bambino Gesù Children’s Hospital IRCCS, Rome, Italy; 3 UCL Institute of Child Health, 30 Guilford Street, LONDON WC1N 1EH, UK; 4 The Liver Unit, Birmingham Children’s Hospital, Birmingham, B4 6NH, UK; 5 Pediatrics Department, CHU Sainte-Justine, 3175 Cote St Catherine, Montreal Quebec, H3T 1C5, Canada; 6 Unit of Hepatobiliary Imaging, Department of Radiology, Bambino Gesù Children’s Hospital IRCCS, Rome, Italy; 7 Reference Center for Inherited Metabolic Diseases, Hôpital Robert Debré, Paris, France; 8 Department of Paediatrics, Section of Paediatric Nephrology, Genetics and Metabolism, University Hospital “Germans Trias i Pujol”, Badalona. Universitat Autònoma de Barcelona, Catalonia, Spain; 9 Ute Spiekerkoetter, Department of Pediatric and Adolescent Medicine, University Children’s Hospital, 79106, Freiburg, Germany

**Keywords:** Hepatorenal tyrosinaemia, Fumarylacetoacetase, Succinylacetone, Nitisinone, Cirrhosis, Acute live failure, Hepatocellular carcinoma, Low tyrosine diet

## Abstract

The management of tyrosinaemia type 1 (HT1, fumarylacetoacetase deficiency) has been revolutionised by the introduction of nitisinone but dietary treatment remains essential and the management is not easy. In this review detailed recommendations for the management are made based on expert opinion, published case reports and investigational studies as the evidence base is limited and there are no prospective controlled studies.

The added value of this paper is that it summarises in detail current clinical knowledge about HT1 and makes recommendations for the management.

## Methodology

This ad hoc working group was chosen from those in Europe and Canada who had the greatest experience and number of patients with HT1. The literature was reviewed and as a result it was decided that it was insufficient to warrant writing formal guidelines and instead only recommendations were written. The first draft was written by the chairman (JVL) and revised in a workshop. Subsequent revisions were circulated and after repeated iterations finally agreed.

## Introduction

**Figure 1:**
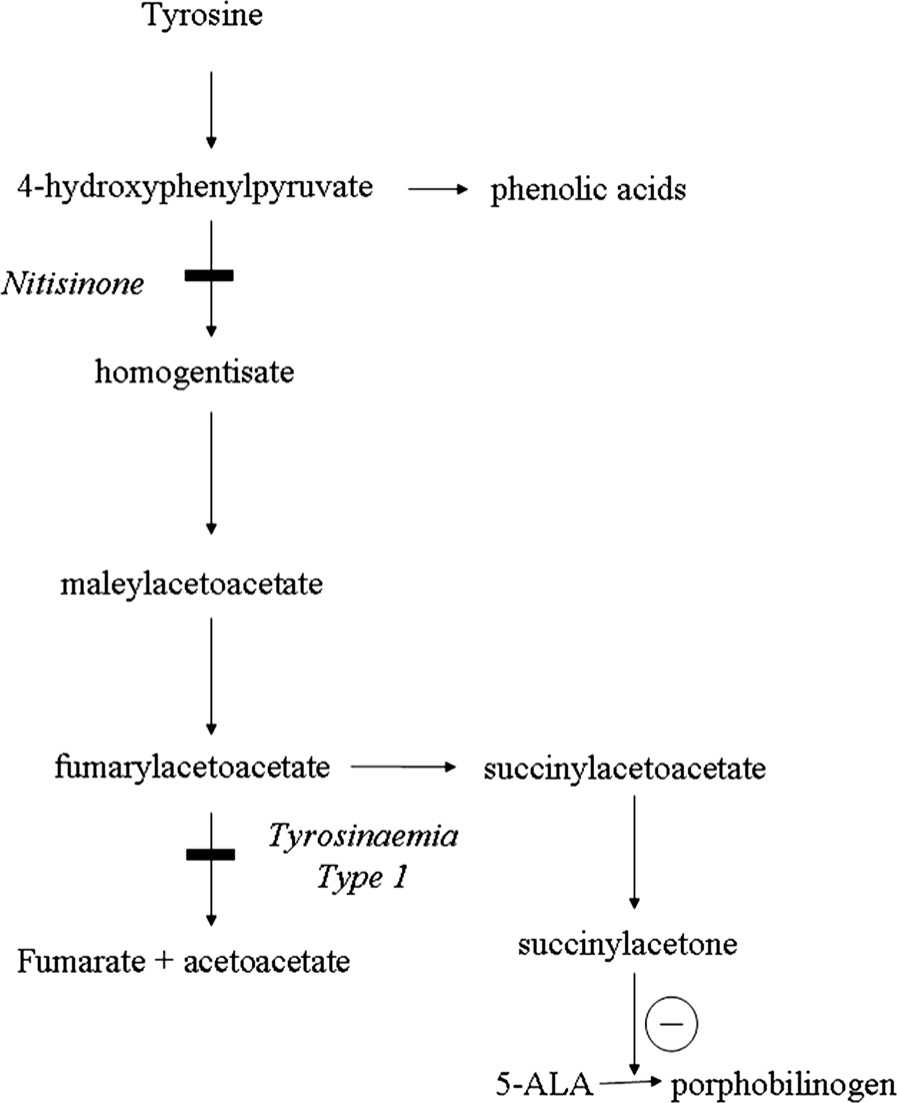
**Tyrosine degradation pathway: HT1 is caused by a defect in the fumarylacetoacetase. **Succinylacetone is a potent inhibitor of 5-aminolaevulinate dehydratase. Nitisinone inhibits 4-hydydroxyphenylpyruvate dioxygenase reducing flux through the pathway.

Tyrosinaemia type 1 (HT1) is caused by a defect in the final enzyme of the pathway of the degradation of tyrosine, namely fumarylacetoacetase (FAH, EC 3.7.1.2, Figure 1). As a result of the metabolic block toxic metabolites are formed including succinylacetone, maleylacetoacetate and fumarylacetoacetate. These are responsible for severe disruption of intracellular metabolism of the liver and kidney. For more details of the biochemistry, molecular genetics and pathophysiology see [1]. HT1 has a birth incidence of approximately 1 in 100,000 in most areas but is more common in some regions, notably in Quebec, Canada [2].

Nitisinone is a compound that has revolutionised the management of tyrosinaemia type 1 [3,4]. It is a potent inhibitor of 4-hydroxyphenylpyruvate dioxygenase, an enzyme that is upstream of fumarylacetoacetase (Figure 1). As a result the flux through the pathway is markedly reduced and in most patients there is a rapid decrease in the concentrations of succinylacetone, an increase in tyrosine and a clear clinical improvement.

The purpose of this article is to review current management and to develop recommendations that should assist with the clinical management of HT1. However there is little evidence base for the management of HT1 so these recommendations are largely based on studies already published [5,6], on expert opinion and unpublished data.

## Clinical presentation and diagnosis

Most patients will present clinically in regions without a specific newborn screening programme for tyrosinaemia type 1 although a few may be identified because of affected siblings or abnormalities of plasma or urine aminoacids. However the latter cannot be relied on and most patients will therefore still present with symptoms.

### Clinical illness

The main organs that are affected are the liver, kidney and peripheral nerves.

#### Hepatic disease

The liver is the organ that is usually most severely affected. Acute liver failure with onset in the first weeks or months of life is common with clotting abnormalities, ascites and oedema secondary to hypoalbuminaemia. Haemorrhage is frequent but jaundice is usually mild and the plasma aminotransferases may be only slightly or moderately elevated. Patients go on to develop cirrhosis, liver nodules and hepatocellular carcinoma. They may also present with any of these at a later age without liver failure. On clinical examination the liver is firm or even hard. Hypoglycaemia may by caused by liver failure and hyperinsulinism [7].

#### Renal disease

The characteristic renal disease is a tubular disorder with a Fanconi syndrome, the severity of which is variable. Although the typical features include aminoaciduria, glycosuria, phosphaturia and renal tubular acidosis, they may not all be present. Patients may develop hypophosphataemic rickets, which can be severe. The renal disease may progress with nephrocalcinosis, glomerulosclerosis and chronic renal failure. Although renal disease may be the predominant feature, there is always some co-existing liver disease of varying severity.

#### Neurological disease

The most characteristic neurological problem is a porphyria-like syndrome usually precipitated by intercurrent infection. The crises, which may be severe, are characterised initially by pain (including abdominal pain mimicking an acute surgical emergency), weakness and autonomic changes such as hypertension. Patients may develop an acute progressive ascending motor neuropathy, often with respiratory distress requiring assisted ventilation.

### Timing of presentation

Patients may present in infancy, childhood or as adults [1,8]. The presentation can be variable, even within one family. The age of presentation broadly correlates with severity.

#### Acute

These patients present before the age of six months, the most severe between 0–2 months [8], usually with a severe liver disease characterised particularly by a synthetic failure. This is the most common presentation. These patients are particularly susceptible to infections [9].

#### Sub-acute

These patients present with progressive liver disease usually also in the first year of life that is usually less severe than the younger patients. The main features are coagulopathy, failure to thrive, hepatosplenomegaly and rickets.

#### Chronic

These patients are more than one year old and present mainly with liver and/or renal disease. The illness may be complicated by cardiomyopathy [10] and neurological problems such as porphyria-like episodes. Cirrhotic liver changes are generally already present.

#### Newborn screening

Newborn screening for HT1 is not widely available because of the rarity of HT1 in most areas. However together with early treatment with nitisinone and diet, it is the medical management of choice [11,12]. Recent studies have shown confirmed this [13] Newborn screening is best performed using succinylacetone (SA) as a primary marker because it is sensitive and specific. Blood spot tyrosine is neither specific nor sensitive. Although biochemical abnormalities may be identified shortly after birth [14], babies with HT1 are rarely symptomatic in the first days of life. Newborn screening enables treatment of children who are not yet clinically ill. However newborn babies diagnosed by screening have markedly raised AFP levels [15] and mild abnormalities of coagulation.

### Investigations

#### Diagnostic tests

The most useful test is for succinylacetone that may be measured in plasma, dried blood spot as used in newborn screening (DBS) or urine. This is highly specific and sensitive. SA may not be detected in routine organic acids analyses in urine, particularly if the concentration of SA very low or the urine very dilute so specific assays may be needed. Testing is urgent if tyrosinaemia type 1 is suspected.

### Baseline tests

Initial tests for HT1 should include the following:

Blood/ plasma

Blood gases

Liver function tests: bilirubin, aspartate and alanine aminotransferase (AST, ALT),

alkaline phosphatase, γ glutamyl transpeptidase (γGT) albumin.

Coagulation: Prothrombin time, partial thromboplastin time, fibrinogen,

Urea and electrolytes, creatinine

Calcium, phosphate

Glucose and ammonia (in acute liver failure)

Full blood count

Aminoacids (quantitative)

α-fetoprotein (AFP)

Succinylacetone (if available. Note: plasma SA is protein bound and is a better test with which to monitor metabolic control than urine SA [16], although urine SA is more widely available at present.

Urine

Glucose

Aminoacids

Tubular re-absorption of phosphate (TRP)

Calcium/creatinine ratio

Albumin, protein, β2-microglobulin

Organic acids and succinylacetone (Note: Routine organic acid analysis may not be sufficiently sensitive)

Interpretation: Initial tests may show evidence of liver disease, usually with a striking disorder of clotting. Plasma amino acids may show raised tyrosine as well as raised methionine, abnormalities that are consistent with any severe liver disease. AFP is usually markedly raised but it is not specific.

#### Imaging

Imaging for hepatic disease is important, particularly at presentation. All patients should have an ultrasound examination of the liver and kidneys initially and thereafter at regular intervals. If nodules are present in the liver further imaging, preferably by MRI, should be done. However there is no single ideal method. To some degree the method chosen will depend on the resources and facilities available.

Ultrasound with high resolution transducers and colour-doppler remains the first modality of choice. It is rapid and safe allowing identification of the echogenicity of the parenchyma and nodular lesions as small as 2 mm in diameter. Colour-doppler can be used to assess the hepatic vessels and portal hypertension. However not all nodules may be detected and the technique is operator dependent.

Multi detector CT scan is quick and enables multiplanar imaging with good spatial resolution of abnormalities in the liver. Contrast may improve the detail, enabling identification of malignant change. However, a risk-benefit analysis is mandatory to avoid radiation in children already at risk of malignancy so it requires repeated evaluation. Sedation is sometimes required

MRI has emerged as the best technique to help in differentiating nodules in children with chronic liver disease, because of its ability to identify different tissues properties. After intravenous gadolinium injection dynamic sequences provide analysis of nodule vascularisation. Diffusion-weighted imaging may help to distinguish well-differentiated hepatocellular carcinomas from benign nodules [17,18], C Dionisi Vici and L Monti unpublished. However it requires some form of sedation in many children.

Conclusion: All patients should have an ultrasound examination of the liver and kidneys. If nodules are present in the liver further imaging, preferably by MRI, should be done.

Bone X-ray (for those with a definite tubulopathy): wrist or chest

## Immediate management

**Figure 2:**
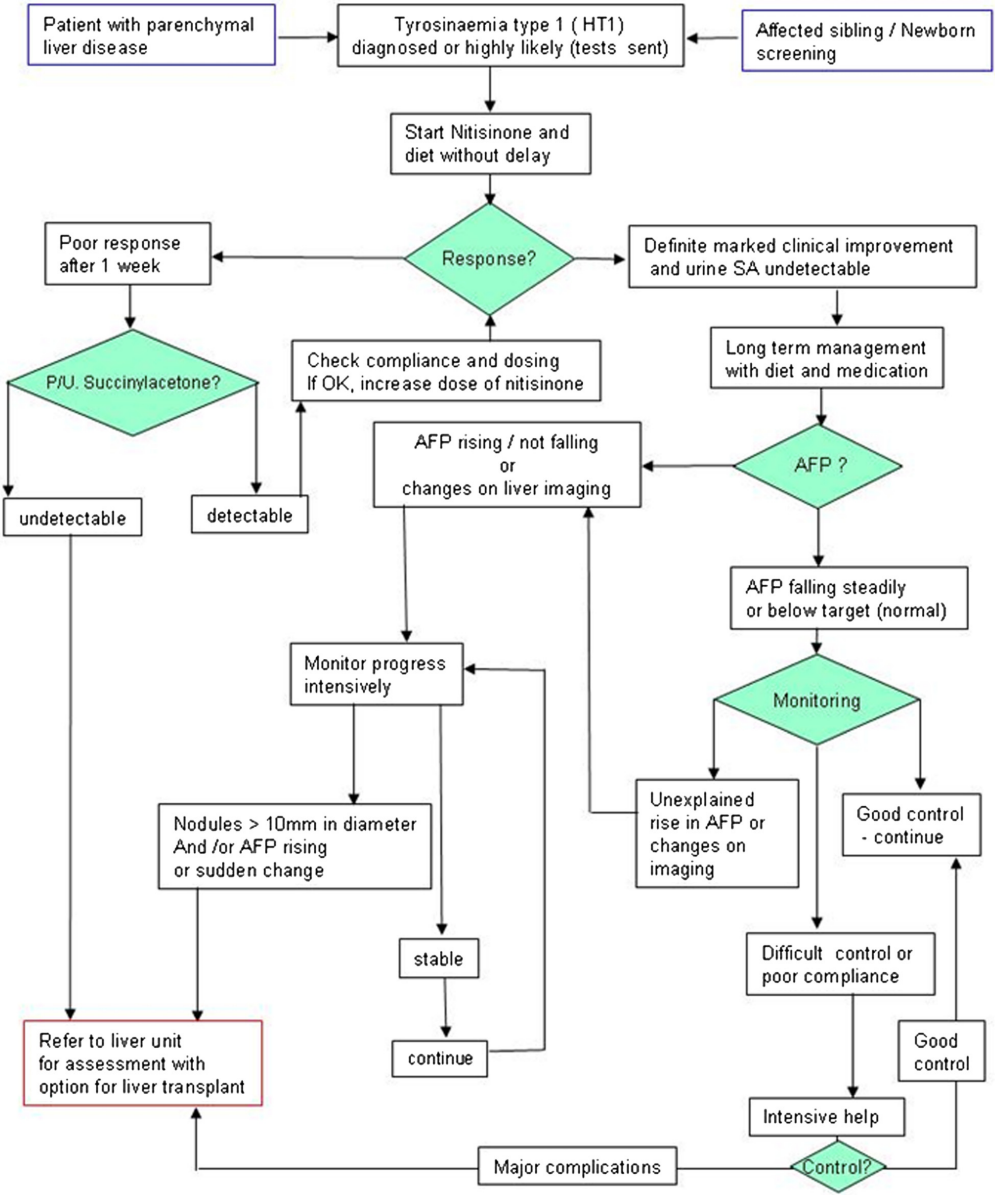
**Outline flow diagram of the management of tyrosinaemia type 1. **This figure must be used in conjunction with the accompanying text.

An outline flow diagram for the management of patients with tyrosinaemia type 1 is shown in Figure 2.

As soon as the diagnosis is confirmed, or even suspected because of liver disease and appropriate investigations have been sent, start nitisinone (NTBC, Orfadin®,) in a dose of 1 mg/kg/d once a day as the half life is 54 hrs [19,20]. A dose of 2 mg/kg/d should be given for 48 hours for those in acute severe liver failure. An alternative approach is to give all patients in liver failure nitisinone at a dose of 2 mg/kg/d from the start and allow the dose to fall with growth to 1 mg/kg/d before increasing it. Nitisinone can only be given orally (or by naso-gastric tube). It is imperative to do so quickly to prevent further liver and kidney damage and avoid potentially major complications such as haemorrhage. The risk of long term complications is also reduced [16]. Details of potential side effects of nitisinone are listed in the appendix.

The patient should also be started on a low tyrosine and low phenylalanine diet. If plasma tyrosine concentrations are raised a high energy diet with a phenylalanine and tyrosine free aminoacid supplement should be given (0.5-1.0 g/kg/d) but natural protein should be introduced early and certainly once plasma tyrosine concentrations are falling. If plasma tyrosine concentrations are initially normal or only slightly raised, natural protein should generally be introduced at the outset. Fructose and galactose from should be excluded until the diagnosis of galactosaemia and fructosaemia have been excluded.

Patients, particularly younger ones, may be seriously ill and require intensive supportive measures including clotting factors albumin electrolytes, correction of acid–base status and aggressive treatment of infections [9]. Children with a plasma bilirubin concentration of more than 100 μmol/l, plasma ammonia of more than 100 μmol/l or with severe acute liver failure at presentation should be discussed with a liver transplant centre at an early stage.

### Monitoring tests

The essential investigations to check the response and to monitor progress are liver function tests, AFP, coagulation studies, quantitative plasma aminoacids and quantitative blood or urine succinylacetone.

### Response to nitisinone

The response to nitisinone is usually rapid. Coagulation usually improves within 48 hours and all patients should respond within a week. Succinylacetone in the urine and blood should no longer be detectable after the first 24 hours.

The concentrations of plasma tyrosine and urine phenolic acids will increase as a result of the inhibition of the 4-hydroxyphenyl pyruvate dioxygenase although the increase in these should be blunted by the diet.

AFP will fall more slowly, usually logarithmically. It there is not a steady fall, check the liver imaging carefully. It can take up to one year or even longer for the AFP to fall to the target value < 10 ng/l.

### Failure to respond

Normally there is a marked improvement in the liver function and coagulation within less than one week but, if the liver function does not normalise or succinylacetone can still be detected in plasma and/or urine, then first check compliance and the dosing. If nitisinone is being given correctly, then the dose should be increased to 2 mg/kg/d, if not already receiving that dose. As the plasma concentrations in individuals are variable [16], if rapid assay of plasma nitisinone level is available it may be useful in detecting problems.

Recovery from severe liver failure is possible but the time taken to do so is variable in severely ill patients. In such patients, management must be individualized during the early days of nitisinone treatment. If the patient deteriorates, as judged by coagulation, liver function (progressive jaundice or hyperammonaemia) or signs of encephalopathy, then orthotopic liver transplantation should be considered.

## Long term management

Before the advent of nitisinone major complications of HT1 including cirrhosis, liver failure, hepatocellular carcinoma and porphyria-like syndrome were frequent. Even when being treated with nitisinone, there are still important risks of long term complications of HT1, most importantly hepatocellular carcinoma. All patients must be followed carefully.

### Medication

Nitisinone must be continued without interruption. Failure to do so may precipitate serious complications including acute liver failure, a neurological crisis [21] or even hepatic malignant change.

On the standard dose of 1 mg/kg/d, the plasma concentrations in individuals that suppress SA are variable [16,20]. Adjustments of the dose based on plasma nitisinone concentrations may be indicated. However the target NTBC concentrations in plasma are uncertain and vary from 30-50 μM [22]. Some prefer to maintain the concentration above 50 μM [23], G Mitchell personal communication. Others use a value greater than 40 μM [C De Laet and P Goyens personal communication]. However lower doses of nitisinone have been found to be effective [24,25]. In one case the serum nitisinone concentration was only maintained above 30 μmol/l [24] with apparently good metabolic control. Whatever the dose, complete suppression of succinylacetone concentrations is essential. Obese patients require a lower dose than that calculated from total body mass. For older children and adults the dose may be calculated using 35 mg/M^2^/day.

### Additional tests (Molecular Genetics)

Molecular genetic studies may be needed for counselling, prenatal diagnosis and family screening. One splice mutation is prevalent in French-Canadians [26] and more than 40 mutations are now known [27,28].

### Clinic appointments and monitoring

The patients should be monitored regularly. The frequency should take account of the age of the patient, the severity of the illness, the family’s understanding and compliance. It is suggested that in the first year the patient is seen every month (or even every week) until the patient is stable and well controlled and the family confident. Thereafter the intervals can be lengthened.

The tests that should be included in each visit are:

Blood/ plasma

Blood gases

Liver function tests: bilirubin, aspartate and alanine aminotransferase (AST, ALT), alkaline phosphatase, γglutamyl transpeptidase (γGT), albumin.

Coagulation: Prothrombin time, partial thromboplastin time, fibrinogen,

Urea and electrolytes, creatinine, calcium, phosphate

Full blood count

Aminoacids (quantitative)

α-fetoprotein (AFP)

Succinylacetone (if available)

Albumin

Nitisinone (NTBC)

If the patient is in acute liver failure plasma glucose and ammonia should be added.

Recently, a simple method allowing the simultaneous determination of NTBC, succinylacetone, tyrosine, phenylalanine, and methionine on a dried blood has been developed with great practical advantages especially in paediatric patients [29].

Urine

Glucose

Aminoacids

Tubular re-absorption of phosphate (TRP)

Calcium/creatinine ratio

Urine acidification (by the locally preferred method)

Albumin, Protein, β2-microglobulin

Organic acids and succinylacetone (note: A specific assay for succinylacetone may necessary to detect very low concentrations as routine organic acid assays may not be sufficiently sensitive).

The following investigations should be done as with any patient on a strict diet at intervals of every 6 months to one year.

Iron and ferritin, vitamins A,D,E, Folate & vitamin B_12,_

Micronutrients (Se, Zn, Cu)

### a-fetoprotein

The AFP should decline steadily and it should be close to the target (normal) value of 10 ng/l by one year but there is considerable variability so that some reach the target earlier and a few later. A slow decrease, failure to normalise or a slight rise should be regarded with suspicion [30] and prompt a careful review of the imaging. The value of lectin reactive-AFP is unclear [31]. If there is any doubt the patient should be referred for assessment by the liver transplant team.

### Diet

The low tyrosine low phenylalanine diet must also be continued indefinitely and should be carefully supervised. The target plasma concentrations of tyrosine and phenylalanine are unclear and long term follow-up studies are urgently needed. The increase in plasma concentrations are associated with increased CSF concentrations of tyrosine [32]. The effects on the tryptophan-derived neurotransmitters [29] is unknown. As a guide the aim should be to keep tyrosine concentrations between 200 – 400 μmol/l up to the age of about 12 years. This is not easy and some centres allow plasma tyrosine concentrations up to 500 μmol/l. After the age of 12 years it is common to allow the plasma tyrosine concentrations to rise modestly but the safety of this is not known and it needs to be kept under careful review. As it is difficult to measure the plasma aminoacids fasting they should be measured under the same conditions each time. To maintain these concentrations of tyrosine, a diet similar to that used in phenylketonuria but without tyrosine supplements is necessary. The quantity of natural protein will be restricted and given as exchanges. A phenylalanine and tyrosine free aminoacid supplement is necessary. Low phenylalanine concentrations may be damaging. If the plasma phenylalanine concentrations are persistently very low, some clinicians prescribe phenylalanine supplements but plasma phenylalanine concentrations show a marked diurnal variation [33,34] and the precise timing of any supplements is unclear. Such phenylalanine supplements may increase plasma tyrosine concentrations. The diet should be monitored as any strict low protein diet.

Whilst the eye complications are rare with tyrosine concentrations below 800 μmol/l (see below) there is increasing concern about the cognitive outcome. Many patients on nitisinone have learning difficulties [35] and it is widely thought that this is associated with the increased tyrosine concentrations. Strict control of plasma tyrosine concentrations is to be encouraged. However a stricter diet is not easy and the evidence that stricter control of plasma tyrosine concentrations improves the outcome is not conclusive [36,37]. The relevance of the concentrations of other large neutral aminoacids, notably tryptophan, is uncertain [32].

### Imaging

The risk of developing HCC is not easily assessed but the later the diagnosis is made the greater the risk of hepatocellular carcinoma (see Table 18.1 in reference 1).Constant vigilance is needed in all patients. Hepatic imaging therefore must be done regularly at least every six months, particularly in the late diagnosed patients.

If the liver is uniform ultrasound may be done every 6 months with MRI (or CT if no alternative) every year. However if there are any changes MRI (or CT if no alternative) should be done without delay. Occasionally nodules are present before nitisinone treatment but regress on repeated imaging following treatment [38]. It may be permissible to do an MRI (or CT if no alternative) every two years in patients detected by newborn screening, started on treatment early and who are stable.

If there are nodules cross sectional imaging (by MRI (or CT if no alternative)) should be done every 3–6 months. It is difficult to assess the potential risk of malignancy in liver nodules so that if a nodule has any characteristics suggestive of HCC or if it persists on repeat imaging (within 3–6 months) in a child with a good metabolic response to treatment, the patient should be discussed with a liver transplantation team.

Imaging should also be done without delay if there is any rise or failure of the expected fall in AFP. Any changes need to be reviewed carefully. If there is a sudden change in a nodule, the development of a new nodule or a nodule is larger than 10 mm in diameter, HCC is likely and the patient should be referred for assessment for liver transplantation. Liver biopsy should be avoided because of the risk of seeding metastases.

Renal imaging: The kidneys should be imaged using ultrasound when the liver is imaged in order to monitor kidney growth and changes in renal parenchyma.

### Psychometric assessment

Psychometric evaluation is important but is of little predictive value before 4 years of age. The facilities for psychometric evaluation are generally in short supply. For most units realistically the first assessment should be before school entry and at intervals thereafter according to the apparent progress and the resources available. Neuropsychological studies may be more helpful that standard IQ tests. School reports can be useful to supplement the assessment.

### Eye examination

Eye complications do not appear to be common [16,39] but periodic eye examination with a slit lamp may be indicated (for example annually). There does not appear to be a clear cut-off of plasma tyrosine concentrations either in man or experimental animals [39,40]. It is not clear whether it is necessary in the asymptomatic child. One approach to minimise clinic appointments is to arrange for an initial eye examination looking for anomalies and then only refer if the child develops symptoms (or if compliance is not good with high plasma tyrosine concentrations).

### Bone mineral density

Bone mineral density should be checked as with any patient on a strict diet. It is particularly important in those with any severe or persisting renal tubular disease.

### Long term management and compliance

Nitisinone and the diet must be continued indefinitely but some families will need intensive support to maintain the treatment.

### The role of liver transplantation

Although the quality of life after a successful liver transplant is usually good, there are serious risks including those of the surgery and lifelong immunosuppression.

The indications for liver transplantation include acute liver failure and malignancy. Young babies may need emergency liver transplantation if they fail to respond to nitisinone. If the recommended medical treatment with nitisinone is not adhered to or is not available, the patient is at risk for acute and chronic complications of HT1. They may be considered for liver transplantation according to the classical criteria established before the availability of nitisinone.

If the diagnosis of HCC without extrahepatic disease is proven or the diagnosis of HCC is suspected because of radiological or serological investigations, the patient should be evaluated by a liver transplantation team urgently.

## Pregnancy

The safety of nitisinone in the treatment in pregnancy, and of the accompanying fetal and maternal hypertyrosinaemia, has not been firmly established. However, three patients on nitisinone have had babies who were normal on examination in the newborn period [41,42], unpublished case report. Early follow-up was also normal.

In affected babies SA is detectable in amniotic fluid in pregnancy and both SA and AFP are raised in cord blood [15]. This suggests that the tyrosine degradation pathway is active *in utero*. In one pregnancy both mother and baby had HT1, the nitisinone not only crossed the placenta freely but suppressed the disease in the fetus [42]. If NTBC is proved to be completely safe it might be given to the mother to prevent damage *in utero.* This approach should be regarded as experimental and subject to a research protocol.

## Management of siblings at risk

HT1 is an autosomal recessive disorder so that there is a 1 in 4 risk of the next sibling being affected. HT1 is a severe disease and prenatal diagnosis is feasible. Although nitisinone has revolutionized the management of the disease, the long-term outcome of the patient remains uncertain. The ethical issues are complex and contentious but it is one widely held view is that termination of the pregnancy can be justified. However the prevailing public and family views and the laws vary widely in countries that may use these recommendations. Detailed discussions between the professionals and the family are essential.

The test is best done by mutation analysis if the genotype of the index case is known. The measurement of succinylacetone concentrations in amniotic fluid is reliable but occasional problems have been reported [43]. Where the mutation is not known, a combined strategy has been suggested [1].

If a baby is known to be at risk but has not had a prenatal diagnosis, cord blood [13] or urine (collected at around 6–12 hours of age) should be sent for urgent analysis of succinylacetone.

## Conclusions

With the introduction of nitisinone the prognosis for those with HT1 has improved greatly. However because newborn screening is only available in a few countries most patients still present clinically. Early treatment with nitisinone and strict diet is essential. Careful long term monitoring and management is required. Prospective, controlled treatment studies are needed to develop evidence-based guidelines for the future management of HT1.

## Endnote (Disclaimer)

The recommendations have been scrutinised carefully but there may still be errors. Furthermore new evidence at any time can invalidate them. No liability whatsoever can be taken as a result of using this information. These recommendations are applicable to most patients but there will be occasions when alternative management is more appropriate. If there is any uncertainty or doubt about the management, the patient should be referred a specialist for a further opinion.

## Appendix

Adverse effects of NTBC

Nitisinone is a remarkably safe and well tolerated medicine. The side effects that are recorded are: *common* (<1/10 - ≥1/100) thrombocytopenia, leucopenia, granulocytopenia, conjunctivitis, photophobia, corneal opacity, keratitis, eye pain: *uncommon* ( <1/100 - ≥1/1000) blepharitis, pruritus, exfoliative dermatitis, and erythematous rash.

## Abbreviations

AFP: α-fetoprotein; DBS: Dried blood spot; FAH: Fumarylacetoacetase (enzyme); GCMS: Gas chromatography mass spectrometry; HCC: Hepatocellular carcinoma; HT1: Tyrosinaemia type 1; NTBC: Nitisinone; SA: Succinylacetone; TRP: Tubular reabsorption of phosphate.

## Competing interests

The development of these recommendations, including organising the workshop in Leeds, UK was supported financially by SOBI (Swedish Orphan Biovitrum) but the company did not contribute to or influence the contents of any version. The company gave access to their files on pregnancy in HT1 and earlier draft recommendations.

CDL: The Paediatric Laboratory received financial support for the determination of nitisinone in dried blood spots

JVL has acted as a consultant to SOBI, has chaired symposia sponsored the company and has received payment for his work.

GM has presented at symposia sponsored by the SOBI

HO: SOBI has paid expenses to attend a workshop at the SFIEM annual meeting.

GPM has received financial support from SOBI in order attend expert’s meetings on Tyrosinemia type 1

The other authors have no other conflicting interests to declare.

## Authors’ contributions

The first draft was written by JVL. The members of the working party all participated in a workshop to discuss the recommendations in detail; all reviewed the drafts and agreed the final version. Dr P Goyens worked with Dr De Laet on the drafts. Comments were also received from Prof W Meersseman (Leuven) and Dr F J van Spronsen (Groningen). All authors read and approved the final manuscript.
